# Perspectives for infrared beamlines in fourth-generation synchrotron facilities

**DOI:** 10.1107/S1600577524003813

**Published:** 2024-04-26

**Authors:** Ferenc Borondics

**Affiliations:** a Synchrotron SOLEIL, RD128, L’Orme des Merisiers, Saint Aubin 91190, France

**Keywords:** fourth-generation synchrotrons, infrared nanospectroscopy, scattering scanning near-field microscopy, nano-FTIR

## Abstract

With several fourth-generation, or diffraction-limited, storage rings and multiple beamlines in operation, the missing range of the spectrum was infrared…until recently.

Fourth-generation synchrotron facility upgrades are upon us, and published results are indeed outstanding. With several fourth-generation, or diffraction-limited, storage rings and multiple beamlines in operation, the missing range of the spectrum was infrared ... until recently.

Infrared (IR) beamlines benefit from the accumulated knowledge of more than a century as IR spectroscopy itself is a well accepted and used laboratory-based technique due to its ability to solve scientific problems from a plethora of research fields. Therefore, IR beamlines have been an integral and productive part of the synchrotron world for many decades implemented at multiple facilities, and have reached a worldwide presence (Miller & Dumas, 2013 ▸). Most of these facilities, recognizing the high added value of synchrotron IR techniques, have already committed to or are looking to raise funds for implementing new IR extractions in the upgraded diffraction-limited storage rings.

While third-generation machines already provide diffraction-limited IR beams (Dumas *et al.*, 2006 ▸), upcoming upgrades will significantly improve electron beam stability and characteristics and thus, through a cascade of developments, we can expect higher signal-to-noise ratios in IR experiments.

The first demonstrated implementation of a fourth-generation infrared facility is the IMBUIA beamline in SIRIUS, at the Brazilian Synchrotron Light Laboratory in Campinas, Brazil. Here, we highlight the paper published in the current issue of *Journal of Synchrotron Radiation* by Santos *et al.* (2024 ▸) describing the performance of a scattering-type scanning near-field optical microscopy (s-SNOM) endstation at the IMBUIA-nano branch. Synchrotron IR nanospectroscopy is one of the most recent synchrotron IR techniques that greatly benefits from the ultrabroadband spectrum and diffraction-limited focus spot of synchrotron IR radiation (Hermann *et al.*, 2013 ▸). At SIRIUS the current IMBUA extraction is implemented with a relatively small extraction angle, around 6 mrad × 6 mrad (H × V). However, the results shown in the paper underline that a very important parameter with s-SNOM is the quality of the focused IR beam spot under the atomic force microscopy tip. This can be well realized with smaller extraction angles, *i.e.* shorter source depth, and using single focal length optics and very high quality wavefronts. Wavefront simulations and measurements as well as near-field spectra (showcased in Fig. 1 ▸), their signal-to-noise and bandwidth, especially towards the high-energy part of the spectrum, highlight the advantage of synchrotron IR beamlines. The plans of the IMBUIA team include the implementation of a wider-angle, higher-flux extraction that will benefit near-field experiments and will be also important when implementing traditional, synchrotron-based diffraction-limited IR spectromicroscopy.

Large-opening-angle extractions, due to the typically smaller diameter of the fourth-generation storage ring vacuum vessels compared with their third-generation predecessors, will present some challenges. To achieve extraction angles similar to third-generation IR beamlines, in the 30–80 mrad range, extraction mirrors need to be placed in very close proximity to the electron beam. The electromagnetic shielding of the small vacuum vessels at increasingly higher energies needs to be taken into account, as explained in detail by Bosch (2002 ▸). Radiation emitted from the complex and densely populated structures of multibend achromat lattices needs to be well understood, and innovative beamline optics will be required to manage wavefronts and recollimate optically deep sources (Moreno *et al.*, 2013 ▸). Still, these issues can be overcome and the example of IMBUIA shows that fourth-generation IR beamlines are off to a flying start. With other scheduled upgrades we are looking at a new era of improved IR spectromicroscopy in both the far- and near-field.

## Figures and Tables

**Figure 1:**
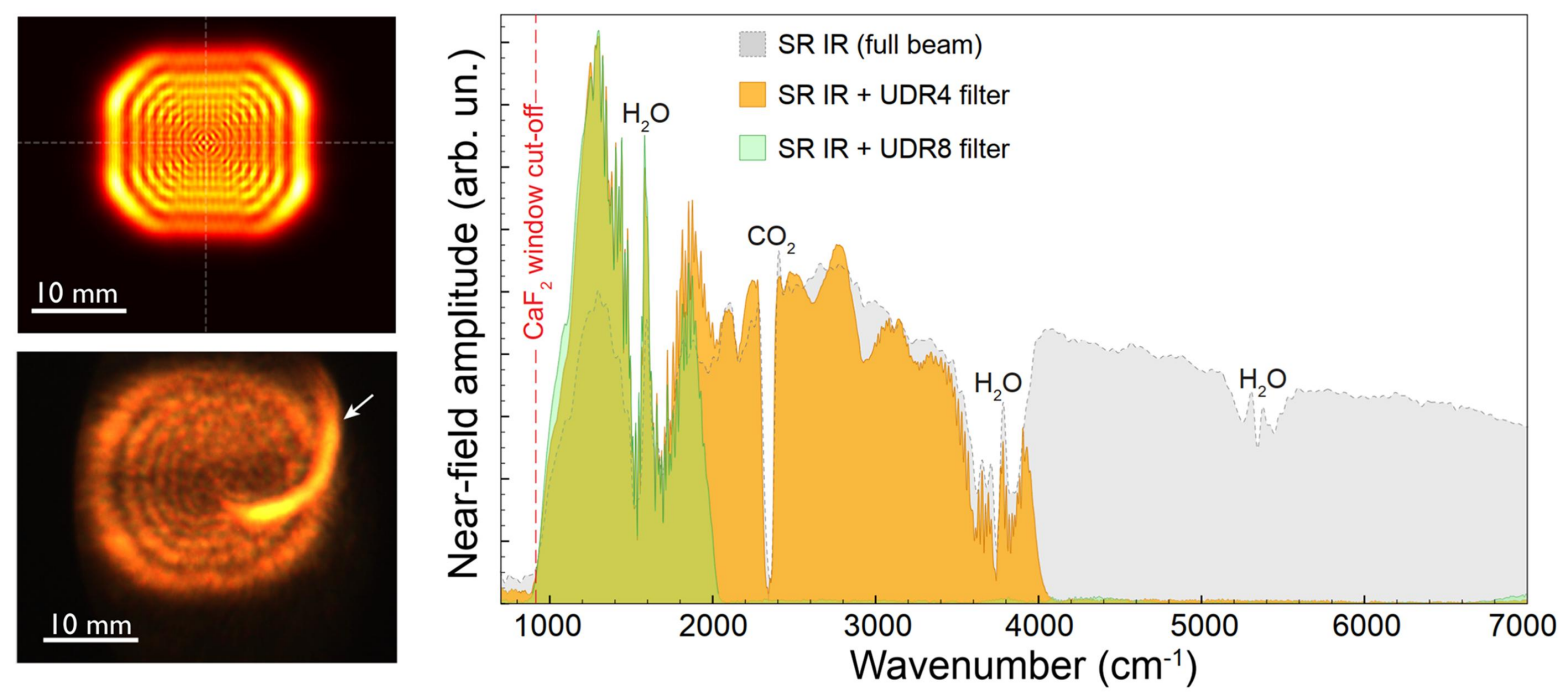
Simulation (top-left) and measurement (bottom-left) of the extraction wavefront of IMBUIA at 633 nm. Broadband near-field nanoFTIR spectrum of the IMBUIA-nano source (right). [Partially reproduced with modifications from Santos *et al.* (2024 ▸) with the permission of the authors.]

## References

[bb2] Bosch, R. A. (2002). *Nucl. Instrum. Methods Phys. Res. A*, **482**, 789–798.

[bb3] Dumas, P., Polack, F., Lagarde, B., Chubar, O., Giorgetta, J. L. & Lefrançois, S. (2006). *Infrared Phys. Technol.* **49**, 152–160.

[bb1] Hermann, P., Hoehl, A., Patoka, P., Huth, F., Rühl, E. & Ulm, G. (2013). *Opt. Express*, **21**, 2913. 10.1364/OE.21.002913 23481749

[bb4] Miller, L. M. & Dumas, P. (2013). *Encyclopedia of Biophysics*, pp. 1106–1112. Berlin, Heidelberg: Springer.

[bb5] Moreno, T., Westfahl, H., Freitas, R. O., Petroff, Y. & Dumas, P. (2013). *J. Phys. Conf. Ser.* **425**, 142003.

[bb6] Santos, T. M., Lordano, S., Mayer, R. A., Volpe, L., Rodrigues, G. M., Meyer, B., Westfahl, H. & Freitas, R. O. (2024). *J. Synchrotron Rad.* **31**, 547–556. 10.1107/S1600577524002364 PMC11075719 38630437

